# Supplementary material for: The causal role of circulating inflammatory markers in osteoporosis: a bidirectional Mendelian randomized study
Source: Front Immunol. 2024 Jul 18;15:1412298. doi: 10.3389/fimmu.2024.1412298 (PMC11291241; doi:10.3389/fimmu.2024.1412298)
Supplement: Supplementary file 1 [file DataSheet_1.zip › supplementary material/CIM-OSTEOPOROSIS /GCST90274793.csv_leave_one_out.pdf]

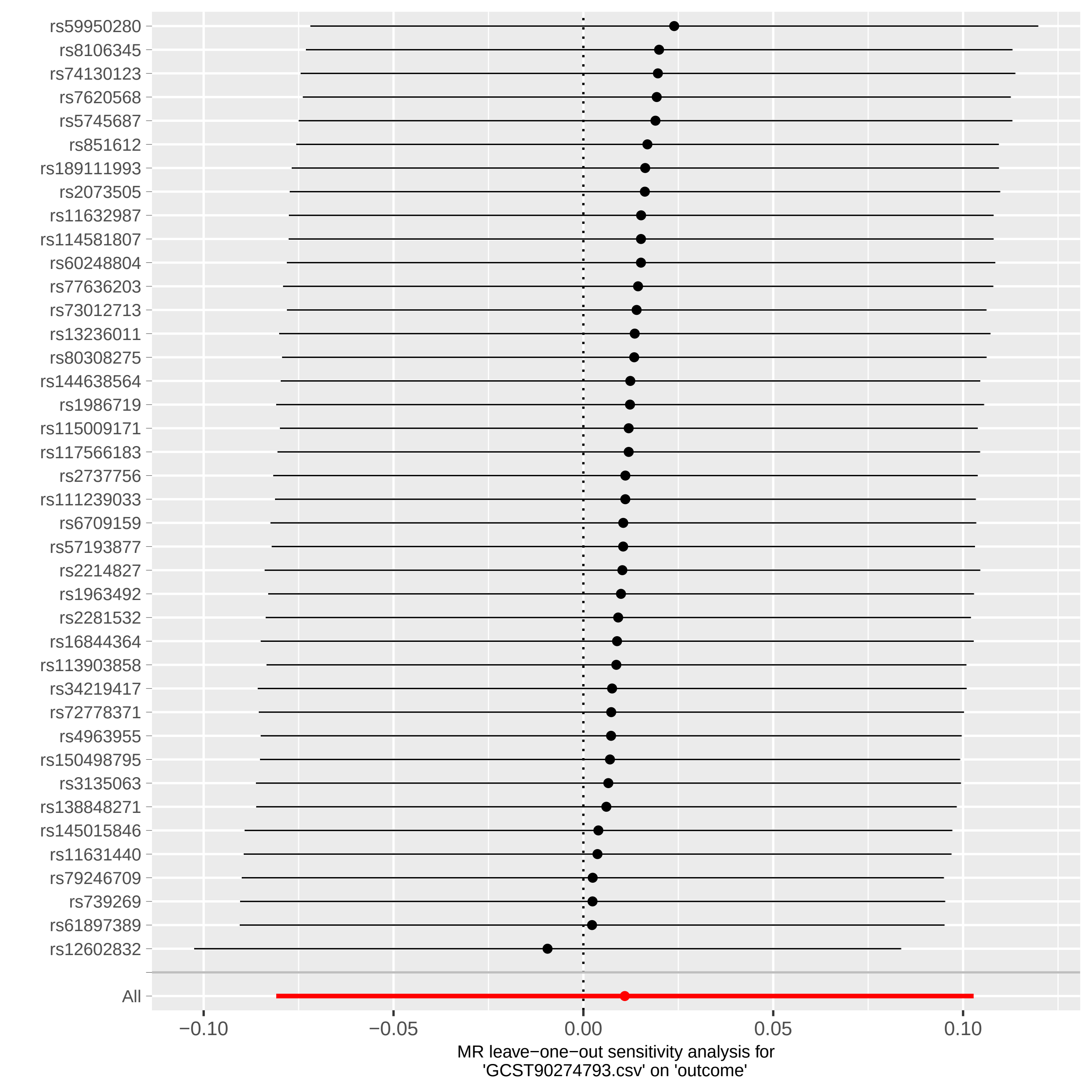
rs59950280
rs8106345
rs74130123
rs7620568
rs5745687
rs851612
rs189111993
rs2073505
rs11632987
rs114581807
rs60248804
rs77636203
rs73012713
rs13236011
rs80308275
rs144638564
rs1986719
rs115009171
rs117566183
rs2737756
rs111239033
rs6709159
rs57193877
rs2214827
rs1963492
rs2281532
rs16844364
rs113903858
rs34219417
rs72778371
rs4963955
rs150498795
rs3135063
rs138848271
rs145015846
rs11631440
rs79246709
rs739269
rs61897389
rs12602832
All
−0.10
−0.05
0.00
0.05
0.10
MR leave−one−out sensitivity analysis for
'GCST90274793.csv' on 'outcome'
